# Harnessing upregulated E-selectin while enhancing SDF-1α sensing redirects infused NK cells to the AML-perturbed bone marrow

**DOI:** 10.1038/s41375-023-02126-1

**Published:** 2024-01-05

**Authors:** Laura Sanz-Ortega, Agneta Andersson, Mattias Carlsten

**Affiliations:** 1 Center for Hematology and Regenerative Medicine, Department of Medicine, Huddinge, Karolinska Institutet, Stockholm, Sweden; 2 Center for Cell Therapy and Allogeneic Stem Cell Transplantation, Karolinska Comprehensive Cancer Center, Karolinska University Hospital, Stockholm, Sweden

**Keywords:** Acute lymphocytic leukaemia, Immunotherapy

## Abstract

Increased bone marrow (BM) homing of NK cells is associated with positive outcome in patients with acute myeloid leukemia (AML) treated within adoptive NK cell transfer trials. While most efforts to further improve the efficacy focus on augmenting NK cell persistence and cytotoxicity, few address their ability to home to the tumor. Here, we decipher how AML growth alters the BM niche to impair NK cell infiltration and how insights can be utilized to resolve this issue. We show that AML development gradually impairs the BM homing capacity of infused NK cells, which was tightly linked to loss of SDF-1α in this environment. AML development also triggered up-regulation of E-selectin on BM endothelial cells. Given the poor E-selectin-binding capacity of NK cells, introduction of fucosyltransferase-7 (FUT7) to the NK cells per mRNA transfection resulted in potent E-selectin binding and stronger adhesion to E-selectin^+^ endothelial cells. Co-introduction of FUT7 and gain-of-function CXCR4 (CXCR4^R334X^) redirected NK cell homing to the BM of AML-bearing mice nearly to the levels in AML-free mice. This work shows how impaired NK cell homing caused by AML-induced microenvironmental changes can be overcome by genetic engineering. We speculate our insights can help further advance future NK cell immunotherapies.

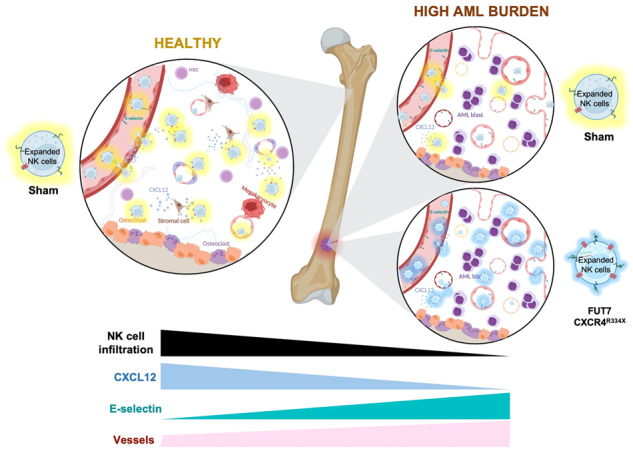

## Introduction

Immunotherapy has rapidly emerged as a treatment option for a broad range of cancers. The use of chimeric antigen receptor (CAR)-T cells is currently reforming how we treat hematological malignancies such as B cell lymphomas, acute lymphoblastic B cell leukemia (ALL), and more recently also multiple myeloma (MM) [1]. Adoptive infusion of natural killer (NK) cells hold promise for the treatment of myeloid leukemias with response rates of up to 50% [2, 3]. Remission in acute myeloid leukemia (AML) and high-risk myelodysplastic syndromes (MDS) have been positively linked to the bone marrow (BM) infiltration potential of the NK cells [3, 4]. Yet, few studies have addressed how to redirect adoptively infused NK cells to the BM compartment [5, 6]

Insights from MM and ALL have uncovered that tumor development in the BM alters the microenvironment. For instance, tumor-mediated suppression of BM stromal cells is reported to suppress SDF-1α levels in this compartment leading to poor infiltration of CXCR4^+^ lymphocytes [7, 8]. Although the SDF-1α levels in the BM can be restored following efficient tumor eradication by chemotherapy [8], this takes weeks to months and is not always possible, especially in the case of refractory disease. While reduced infiltration of adoptively infused lymphocytes has not been addressed in myeloid leukemia, it is well known that AML development triggers increased E-selectin expression on surrounding endothelial cells in the BM niche causing chemorefractoriness and AML dormancy [9, 10].

Here we show that AML development in the BM suppresses SDF-1α levels and significantly reduces the degree of infiltration by adoptively infused NK cells in a xenogeneic mouse model. We demonstrate that human NK cells electroporated with mRNA coding for fucosyltransferase-7 (FUT7) results in durable fucosylation of E-selectin ligands and thereby strong adhesion to E-selectin^+^ endothelial cells. Utilizing this insight to harness the selectively upregulated E-selectin on endothelial cells in the AML BM niche, together with overexpression of gain-of-function (GoF) CXCR4, CXCR4^R334X^, we show that the significant reduction of NK cell BM infiltration in animals with high AML burden is almost restored to that of non-tumor-bearing animals. Overall, these results establish that AML growth in the BM suppresses the infiltration of adoptively infused NK cells via reduced levels of SDF-1α. Based on this and the known upregulation of E-selectin observed on BM vessels adjacent to the leukemic cell [9, 11–13], we have developed a novel approach to efficiently redirect NK cells to the AML niche, which may be explored to improve response rates of adoptive NK cell immunotherapy against myeloid leukemia.

## Methods

### Isolation and expansion of NK cells

Peripheral blood cells were collected from healthy blood donor buffy coats (Ethical approval Dnr 2006/229-31/3). Peripheral blood mononuclear cells (PBMCs) were isolated by high-density gradient centrifugation before being cryopreserved. NK cells were isolated from thawed PBMCs using the human NK cell isolation kit (Miltenyi, Bergisch Gladbach, Germany) for magnetic bead separation according to the manufacturer’s protocol. Ex vivo expansions were performed as previously described [14]. Briefly, isolated NK cells were co-cultured with irradiated SMI‐LCL feeder cells at a ratio of 1:20 in X‐VIVO 20 cell culture medium (Lonza, Walkersville, MD, USA) containing 10% heat‐inactivated human AB serum (Invitrogen), 2 mM GlutaMAX‐1 (Gibco, Waltham, MA, USA) and 500 IU mL^−1^ IL‐2 at 6.5% CO_2_ and 37 °C. Fresh media was supplied to cells starting on day 4 of expansion, and then, cells were subcultured every 2–3 days until they were harvested for the experiments.

### Murine xenograft tumor model

Animal experiments were performed under ethical approval (ID1533 and 22248-2022) by Jordbruksverket, Sweden. NSG-SGM3 female mice aged 2 to 5 months were used for this study. Male mice were used only when indicated. The AML mouse model was generated by intravenously (i.v.) transplanting 0.5 million HL-60 cells into NSG-SGM3 mice and the AML engraftment was evaluated in the organs by flow cytometry at different timepoints after AML cell injection. For this, tissues were mechanically dissociated, and HL-60 cells were identified by expression of human CD45 and human CD33. AML engraftment is presented as % HL-60 cells within the alive population in each tissue. For endothelial cell stains, we collected all the BM endothelial cells (BMECs) as previously described [15, 16]. Briefly, bones (femur and tibia) were mechanically dissociated in PBS supplemented with 2% FBS (Gibco) and the marrow cells were collected. The cells within remaining bone fragments were also obtained following treatment with collagenase II (ThermoFisher, Waltham, MA, USA) (1 mg/mL in MEM 10% FBS) for 60–90 min under gentle agitation at 37 °C. Both cell fractions were pooled and resuspended in PBS 2% FBS before being analyzed by flow cytometry. BMECs were identified as mouse CD45^-^ LIN^-^ mouse CD31^+^ cells within the live population. Finally, in some experiments, sternums and BM supernatants were also collected for additional analyses of the AML-BM niche, using immunohistochemistry and ELISA analyses, respectively.

### Immunohistochemistry (IHC) analysis

Mouse bones (sternums) were fixed in 4% paraformaldehyde (PFA) for 48 hours and decalcified in EDTA for 72 hours, before paraffin embedding and mounting 4 *μ*m sections on slides. After deparaffinization, heat-based antigen retrieval, endogenous peroxidase blockade using 3% hydrogen peroxide, and blocking were performed. Anti-COX IV (3E11) and anti-mouse CD31 (D8V9E) rabbit monoclonal antibodies (Cell Signaling Technology, Danvers, MA, USA) were used as primary antibodies for identification of HL-60 cells and mouse endothelial cells, respectively. The primary antibodies were incubated overnight at 4 °C. Detection was performed using the Vectastain ABC-HRP Kit (Vector Laboratories, Burlingame, CA, USA) with diaminobenzidine (DAB; Vector Laboratories). Counterstaining was performed using hematoxylin. The stained tissue sections were scanned using a 3D Histech Slide Scanner and Pannoramic MIDI (3D HISTECH) and the Pannoramic Viewer (3D HISTECH) was used for taking images of the sections at different magnifications. The substitution of the primary antibody for serum and/or staining of healthy BM (in the case of COX IV staining for tumor detection) were used as negative controls to detect specific staining.

### NK cell transfection

4 μg/10^6^ cells of mRNA were transfected into ex vivo expanded NK cells using the MaxCyte GT electroporation instrument (MaxCyte Inc., Gaithersburg, MD, USA). mRNAs encoding for human FUT7 were prepared from a plasmid purchased from SinoBiological (Beijing, China): using the HiScribe T7 ARCA mRNA Kit (with tailing) (New England BioLabs, Ipswich, MA, USA) and the MEGAclear™ Kit (Invitrogen) according to the manufacturer’s instructions. Custom-made mRNAs encoding for human FUT7 and CXCR4^R334X^ were obtained from TriLink Biotechnologies.

### Cellular homing assays in vivo

Sixteen hours after FUT7 mRNA electroporation or two hours after CXCR4^R334X^ mRNA electroporation, 10 × 10^6^ NK cells were injected i.v. into healthy (tumor-free) NSG-SGM3 mice or mice with either low or high tumor burden. Animals received 2 × 10^5^ IU of IL-2 intraperitoneally immediately after injection of the NK cells and every 24 hours. Peripheral blood, BM, spleen, liver and lungs were harvested from animals 24 or 48 hours after cell transplantation. Tissues were dissociated and the homing of NK cells were examined by flow cytometry based on human CD45 and human CD56 expression. NK cell infiltration is presented as % NK cells within the alive population in each tissue. A non-transplanted mouse was included as a negative control in each analysis.

### Statistical analysis

Statistical analyses were performed using Prism (GraphPad Software Inc, San Diego, CA, USA). The Wilcoxon signed-rank test was used to assess significance in paired non-parametric datasets and the Mann-Whitney U test was used for unpaired non-parametric datasets. Significant results were marked by *p < 0.05, **p < 0.01, ***p < 0.001 and ****p < 0.0001 while non-significant results were not further specified.

### Additional methods

See the Supplemental Material for additional material and methods.

## Results

### Myeloid leukemia growth disrupts the BM microenvironment and impairs NK cell infiltration

**Fig. 1:**
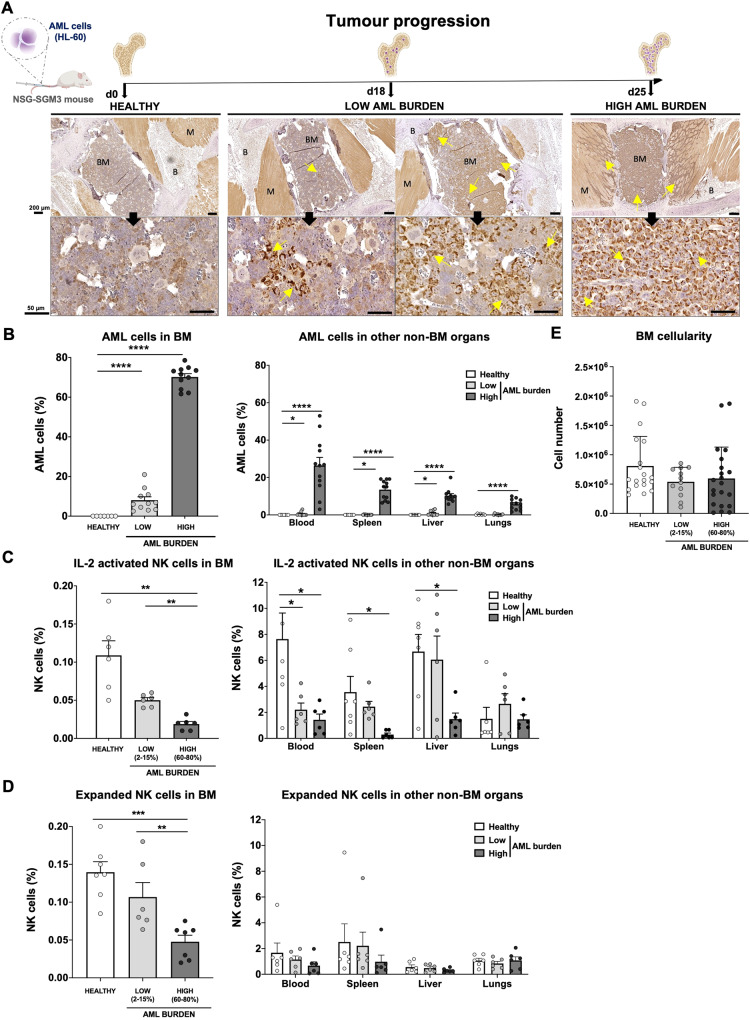
AML development disrupts the BM microenvironment and impairs NK cell infiltration. **A** Experimental design of the in vivo AML model showing different stages of tumor development in the BM. Representative IHC stainings of COX IV protein in BM mouse samples from healthy (tumor-free), low and high tumor burden conditions. Arrows indicate COX IV^+^ cells (AML blasts) present within the central BM as well as spreading to other regions. Scale bar: 200 µm (upper panels), 50 µm (bottom panels). Legend: BM (bone marrow), **B** (bone) and M (muscle). Created with BioRender.com. **B** Tumor burden in BM and several organs at different stages of AML progression after infusion of HL-60 cells into NSG-SGM3 mice, assessed by FC (*n* = 12 mice/group). Infiltration of **C** IL-2 activated and **D** expanded human NK cells in BM (left panel) and other organs (right panel) 24 hours after infusion comparing healthy (tumor-free), low and high tumor burden conditions, assessed by FC (*n* = 6–7 mice/group). Bars, mean. Error bars, SEM. **E** Total BM cell number recorded by flow cytometry after equal tissue handling and processing, comparing healthy (tumor-free), low and high tumor burden conditions. The Mann-Whitney U test comparing healthy (tumor-free), low and high tumor burden conditions was used for statistics.

To address if the development of myeloid leukemia alters the BM microenvironment and if that impairs lymphocyte infiltration, we established a robust xenograft AML mouse model. The human myeloid leukemia cell line HL-60 was chosen as it is known to establish well but grow relatively slow in NSG-SGM3 mice [17] but also because our data show it is relatively resistant to our NK cells both in vitro and in vivo (Supplemental Fig. 1), allowing us to assess NK cell homing without altering the AML burden post-NK cell infusion. As shown in Fig. 1A, B, i.v. inoculation of the HL-60 cell line into NSG-SGM3 mice resulted in low AML burden (around 2–15%) in the BM at day 18 and high AML burden (60–80%) at day 25. At the later timepoint, AML cells could also be detected in blood (26 ± 4%) and other non-BM organs such as spleen, liver, and lungs. Importantly, the animals were clinically unaffected at either of these two timepoints. Using our model, we next assessed the ability of i.v. infused human NK cells to infiltrate the BM. As shown in Fig. 1C, D, high AML burden resulted in poor BM infiltration of both IL-2 short-term activated and ex vivo expanded NK cells. Importantly, the proportion of NK cells was not influenced by changes to the total cell number in the BM as this was similar between all three conditions (Fig. 1E). The presence of NK cells in other organs such as blood, spleen and liver was also reduced when a significant amount of AML cells were present while this was not observed in the lungs that had the lowest AML burden of all organs assessed (Fig. 1C, D). These data show that AML development in the BM gradually hinders NK cell infiltration, similarly to what has been shown for lymphocytes in the context of ALL and MM [7, 8].

**Fig. 2:**
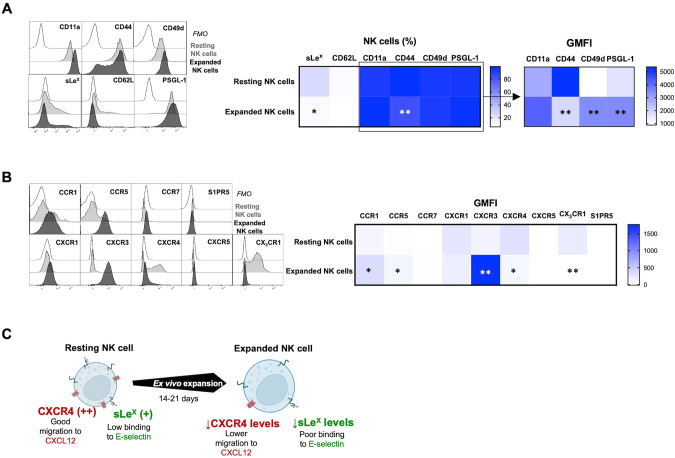
Expanded human NK cells have limited BM homing due to low CXCR4 and sLe^X^ expression. Representative histograms of relevant (**A**) adhesion molecules and (**B**) chemokine receptors on human NK cells before and after expansion and the corresponding heat maps of protein expression (NK cells (%) and/or GMFI) assessed by FC (*n* = 6). **C** Schematic representation showing the limited BM homing potential of NK cells after expansion. The Wilcoxon matched‐pairs signed‐rank test comparing human NK cells before and after expansion was used for statistics. Created with BioRender.com.

A key factor for BM homing is the proper fucosylation of extracellular proteins (such as PSGL-1) on NK cells, which is needed for E-selectin binding and their subsequent rolling on vascular surfaces, which is one of the first steps in the leukocyte adhesion cascade [18]. Moreover, as previously reported, commonly used methods to prepare NK cells for adoptive infusion can alter the chemokine receptor expression and thereby modulate their ability to migrate toward certain chemokines in vitro [19, 20]. Therefore, we next assessed the expression of key chemokine receptors and adhesion molecules on NK cells before and after ex vivo expansion. This revealed low fucosylation levels on ex vivo expanded NK cells as measured by sialyl-Lewis-X (sLe^x^) expression (Fig. 2A), indicating poor binding to E-selectin. Our data also corroborate previous data [19] showing a reduction in the CXCR4^+^ NK cell population (Fig. 2B) following ex vivo expansion. As observed in Fig. 2A, B and summarized in Fig. 2C, the altered selectin ligands and chemokine receptor expression on ex vivo expanded NK cells (Fig. 2C) collectively suggest dampened BM homing capacity.

### Expanded NK cells transfected to express fucosyltransferase-7 have increased sLe^x^ levels resulting in increased endothelial adherence under shear stress

**Fig. 3:**
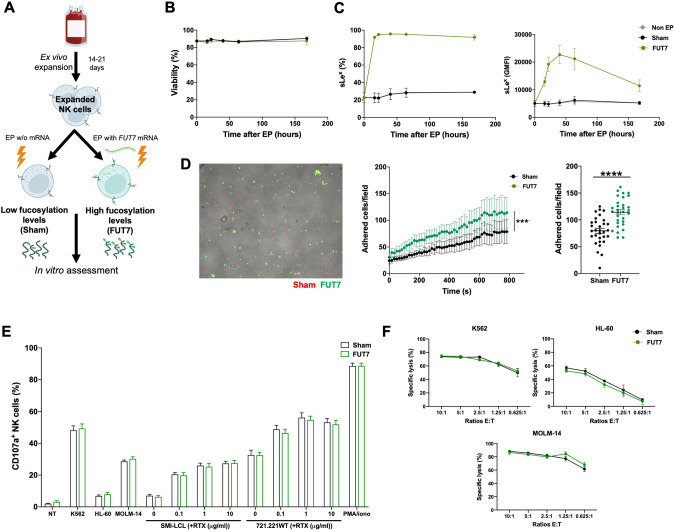
FUT7^+^ expanded human NK cells present high fucosylation levels, increased adhesion to TNFα-stimulated HUVECs under shear stress and maintain their viability and cytotoxic potential. **A** Experimental layout for electroporation of expanded human NK cells with mRNA coding for FUT7. Created with BioRender.com. **B** Viability of ex vivo expanded NK cells from healthy donors following electroporation with 4 μg of FUT7 mRNA per million cells, and **C** sLe^X^ expression levels (% and GMFI) measured by HECA-452 binding. Sham (no mRNA) (*n* = 12). **D** Representative capture of a flow chamber assay movie showing sham (red) and FUT7^+^ (green) NK cells adhered to the TNFα-stimulated HUVEC under physiological shear stress and their in vitro adhesion comparing sham (non-mRNA) and FUT7 mRNA electroporated expanded NK cells (*n* = 6). **E** NK cell degranulation and **F** target cell killing following co-cultures with NK cells and the denoted target or without target (no target, NT) performed 20-24 hours after electroporation of the NK cells with FUT7 mRNA (*n* = 4–6). Bars, mean. Error bars, SEM. The Wilcoxon matched‐pairs signed‐rank test comparing mRNA electroporated vs sham electroporated NK cells was used for statistics.

Given the low cell surface fucosylation levels displayed by ex vivo expanded NK cells, we next wanted to address if increased levels of fucosylation would lead to better adherence to endothelial cells. This que is an early key event in the extravasation cascade [18]. Therefore, we transiently introduced FUT7, the enzyme responsible for synthesizing sLe^X^, to expanded NK cells per mRNA transfection (Fig. 3A). This rapidly increased sLe^X^ on almost all cells for at least one week without impacting viability or the expression of key adhesion molecules (Fig. 3B, C and Supplemental Fig. 2). When challenged with shear stress in flow chamber assays, FUT7 transfected NK cells showed increased adhesion to a monolayer of TNF-α-activated HUVEC cells (Fig. 3D). Importantly, this modification did not alter NK cell function as measured by degranulation and killing of myeloid leukemia and EBV-LCL cell lines (Fig. 3E, F).

### FUT7-modified expanded NK cells distribute differently in vivo compared to control NK cells in tumor-free mice with a trend for more NK cells in the BM

**Fig. 4:**
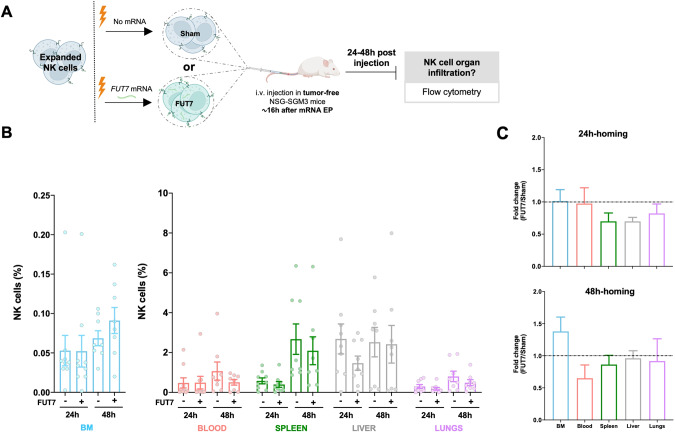
Introduction of FUT7 in expanded human NK cells does not provide a significant benefit in BM homing in tumor-free mice. **A** Experimental layout for in vivo homing of adoptively infused expanded human NK cells electroporated with mRNA coding for FUT7 into healthy NSG-SGM3 mice. Created with BioRender.com. **B** NK cell infiltration (%) in several organs 24 h and 48 h after NK cell transfer, assessed by FC (*n* = 9/group). **C** Comparison of NK cell infiltration in several organs in terms of fold change relative to sham (no mRNA) condition. Bars, mean. Error bars, SEM. The Mann-Whitney U test comparing mRNA electroporated vs sham electroporated NK cells at each timepoint (24 h or 48 h) was used for statistics.

In contrast to settings of stress such as induced during tumor development, E-selectin is constitutively lowly expressed on selected endothelial cells in the body, including in the BM [21, 22]. We therefore first wanted to explore if FUT7-modified NK cells had an increased potential to home to the BM of healthy tumor-free mice (Fig. 4A), similar to as shown for ex vivo fucosylated (exofucosylated) CAR T cells [23]. Following i.v. injection of FUT7-modified NK cells into healthy tumor-free NSG-SGM3 mice, we observed an altered in vivo distribution over time compared to control NK cells (Fig. 4B, C). Although there was a trend for more FUT7-engineered NK cells in the BM 48 hours after injection compared to control, this was not statistically significant. In fact, increased BM homing of FUT7-engineered NK cells was not always the case and highly varied between individual mice and donors (Fig. 4C), likely due to factors such as different basal E-selectin levels on the BMECs and different basal fucosylation levels of each donor. Hence, this data did not clearly indicate that FUT7-modified NK cells have a higher propensity for BM homing compared to controls when infused into tumor-free mice. We speculate that this observation is linked to the generally low E-selectin levels on the endothelial cells in the BM under homeostatic conditions.

### AML progression gradually increases vascularization and E-selectin expression in the BM resulting in increased infiltration of FUT7-modified expanded NK cells

**Fig. 5:**
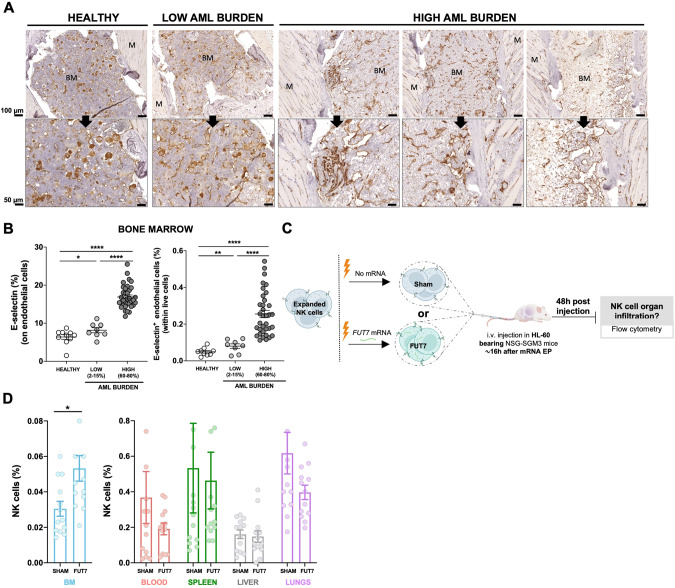
AML progression promotes angiogenesis and E-selectin upregulation on BM endothelial cells, creating a more favorable microenvironment for FUT7^+^ expanded NK cells to infiltrate the BM. **A** Evaluation of changes in the BM endothelium (CD31 expression) during AML progression by IHC. Scale bar: 100 µm (upper panels), 50 µm (bottom panels). Legend: BM (bone marrow) and M (muscle). **B** Assessment of E-selectin expression (%) on the BM endothelial cells and quantification of total E-selectin^+^ BM endothelial cell number by FC. Bars, mean. Error bars, SEM. The Mann-Whitney U test comparing healthy (tumor-free), low and high tumor burden conditions was used for statistics. **C** Experimental layout for in vivo homing of adoptively infused expanded human NK cells electroporated with mRNA coding for FUT7 into (HL-60)-bearing NSG-SGM3 mice. Created with BioRender.com. **D** NK cell infiltration (%) in several organs 48 h after NK cell transfer, assessed by FC (*n* = 12/group). Bars, mean. Error bars, SEM. The Mann-Whitney U test comparing mRNA electroporated vs sham electroporated NK cells was used for statistics.

AML cells are reported to stimulate endothelial cells to upregulate E-selectin and via this interplay become dormant and chemorefractory [9, 10]. Hence, as E-selectin levels on BM endothelial cells are reported to increase during AML development [11], we next assessed if this was also observed in our model. As shown in Fig. 5A, B and Supplemental Fig. 3, engraftment of HL-60 in NSG-SMG3 mice resulted in increased vascularization and E-selectin expression in the BM. The increased density and size of the vessels, as well as a more chaotic vessel organization, could be observed in patches associated with leukemia growth (Supplemental Fig. 3A). Given this data, we next addressed if FUT7-modified NK cells had improved BM homing capacity over control NK cells when infused into NSG-SGM3 mice with high AML BM burden (Fig. 5C). As shown in Fig. 5D, introduction of FUT7 resulted in significantly better BM infiltration compared to controls, while less frequently observed in blood, lungs and spleen. Hence, this data indicates that FUT7-mediated upregulation of sLe^X^ on the NK cells can help them better infiltrate leukemia-containing E-selectin high BM compartments and thereby partially overcome the poor infiltration observed for unmodified NK cells. The role for the E-selectin/fucosylation axis in our model was confirmed by infusion of unmodified NK cells from two donors with distinct baseline fucosylation levels into animals with natural gender differences in E-selectin upregulation following AML BM engraftment (Supplemental Fig. 4). Overall, we conclude that FUT7-modified NK cells significantly better home to the BM of AML-bearing mice due to the combination of increased fucosylation of the NK cells along with leukemia-induced E-selectin upregulation on BMECs.

### Co-introduction of FUT7 and CXCR4^R334X^ in expanded human NK cells nearly restores NK cell BM homing in AML-bearing mice

**Fig. 6:**
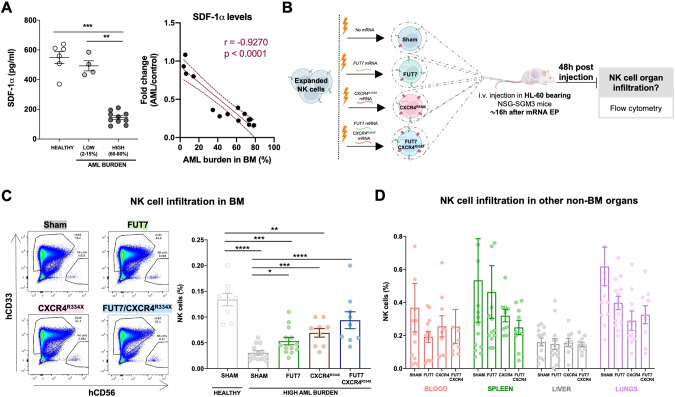
Introduction of FUT7 and CXCR4^R334X^ in expanded human NK cells nearly restores NK cell BM homing in AML-bearing mice. **A** SDF-1*α* BM levels during AML progression, assessed by ELISA and the correlation between tumor burden and SDF-1*α* BM levels. Bars, mean. Error bars, SEM. The Mann-Whitney U test comparing healthy (tumor-free), low and high tumor burden conditions was used for statistics. **B** Experimental layout for in vivo homing of adoptively infused expanded human NK cells electroporated with mRNAs coding for FUT7 and/or CXCR4^334X^ into (HL-60)-bearing NSG-SGM3 mice. Created with BioRender.com. **C** NK cell infiltration in BM in tumor-free and tumor-bearing mice after mRNA electroporation, assessed by FC. Gating strategy for HL60 cells (live^+^hCD33^+^hCD45^+^) and NK cells (live^+^hCD56^+^hCD45^+^)^,^ and representative dot plots from one mouse in each group showing increasing NK cell infiltrations. **D** In vivo homing of ex vivo expanded human NK cells in several organs 48 h after cell transfer, assessed by FC (*n* = 9–15 mice/group). Bars, mean. Error bars, SEM. The Mann-Whitney U test comparing mRNA electroporated vs sham electroporated NK cells was used for statistics.

A reduction in SDF-1α expression has been shown to be one of the main causes for the impaired homing of cytotoxic lymphocytes to MM or ALL containing BM compartments [7, 8]. Recently, SDF-1α has been also found significantly down-regulated in AML [24, 25] Here we show, for the first time, that SDF-1α levels in the BM of NSG-SGM3 gradually drop with increasing AML growth (Fig. 6A). Given our recent insights that introduction of the GoF variant of CXCR4, CXCR4^R334X^, to NK cells results in that they also respond to low levels of the SDF-1α [19], we hypothesized that CXCR4^R334X^ overexpression would help them better infiltrate AML-containing BM compartments with low SDF-1α levels. For this reason, we transiently introduced CXCR4^R334X^ alone or in combination with FUT7 per mRNA transfection to expanded NK cells and evaluated their ability to home to the BM and other tissues of HL-60 engrafted NSG-SGM3 mice (Fig. 6B). Due to the transient CXCR4^R334X^ upregulation following mRNA transfection [19], we performed sequential electroporations to match the optimal expression of both transgenes. As shown in Supplemental Fig. 5A, B, introduction of the mRNAs alone or in combination did not affect NK cell viability while quickly and significantly increasing the fucosylation level and/or CXCR4^R334X^ expression. Importantly, introduction of CXCR4^R334X^ into FUT7-modified NK cells did not cause any alterations in the fucosylation kinetics while expressed to the same levels with the same efficacy. Moreover, NK cell cytotoxicity was unaffected in all conditions as assessed by co-cultures with both myeloid leukemia and EBV-LCL cell lines in the absence or presence of rituximab (Supplemental Fig. 5C, D). This was also the case for the expression of an array of cell surface molecules that remained unaffected (Supplemental Fig. 5E), corroborating previous reports showing mRNA electroporation does not infer major significant changes to the NK cell phenotype [19, 26]. When infused into NSG-SGM3 mice with high AML tumor burden (Fig. 6B), FUT7/CXCR4^R334X^-modified NK cells showed strong BM homing capacity compared to unmodified control NK cells (0.094% vs 0.030%) (Fig. 6C), approaching the potential of unmodified NK cells infused into healthy tumor-free mice (0.094% vs 0.14%). In contrast to NK cells modified with either FUT7 or CXCR4^R334X^ that both had statistically significantly reduced infiltration capacities compared to unmodified NK cells infused into tumor-free mice, FUT7/CXCR4^R334X^-modified NK cells had a restored infiltration capacity of around 67% which was statistically insignificant from the control. Remarkably, the degree of NK cells was reciprocally reduced in several other non-BM organs, with a close inverse association between the infiltration level of NK cells in the BM versus the spleen (Fig. 6D). Interestingly, when infused under healthy conditions, the effect of these modifications was not as significant as in the high AML-bearing condition (Supplemental Fig. 6).

## Discussion

Whether myeloid leukemia development negatively affects the BM homing ability of adoptively infused NK cells has remained unknown. Insights could critically impact on the formulation of future NK cell products to further sharpen their efficacy. We here for the first time demonstrate that AML growth directly impairs the BM infiltration capacity of infused NK cells. As AML progression was shown to gradually reduce BM SDF-1α levels, recapitulating what is observed in patients [24, 25], while increasing E-selectin^+^ BMECs, we explored the potential of modifying ex vivo expanded human NK cells to express FUT7 for intrinsic forced fucosylation along with GoF CXCR4^R334X^ to better sense suppressed SDF-1α levels. These modifications redirected NK cell homing to the AML-containing BM via enhanced binding of upregulated E-selectin and distinct response to the low SDF-1α levels. Our data highlight the relevance of studying how AML-mediated perturbations impact BM homing of infused cell therapy products and how such perturbations can be utilized to more specifically redirect effector cells to the myeloid leukemia niche in vivo.

NK cell presence in the BM after adoptive infusion into patients with myeloid leukemia positively correlates with outcome [4]. Indirect data also show that AML and high-risk MDS patients receiving NK cells with higher CXCR4 expression have a higher likelihood of responding compared to those receiving an NK cell product with lower expression [3]. These data underscore the key role of proper NK cell homing to the site of myeloid leukemia to induce good clinical responses in settings of adoptive cell transfer. However, at the same time, studies have highlighted the relatively poor baseline BM homing potential of infused NK cells [27]. Our recent work has shown that NK cells manipulated to overexpress CXCR4^R334X^ have enhanced BM homing capacity when infused into healthy tumor-free NSG mice compared to control NK cells [19]. We have also shown that infusion of ex vivo expanded human NK cells mRNA transfected to transiently express CXCR4^R334X^ significantly better target AML in the BM of NSG-SGM3 mice resulting in prolonged survival compared to mice treated with unmodified NK cells [28]. This mechanistically and more formally establishes a key role for proper NK cell homing to the BM via CXCR4 for treating myeloid leukemia. Nevertheless, these data were generated using a different and more aggressive tumor model than used in the current work. Due to the aggressiveness of the MOLM-14 cell line used in those mice, we started treatment three days after i.v. inoculation of the tumor cells in that model and although the infused MOLM-14 cells clearly established in the BM, the AML cells did not have enough time to induce perturbations to prevent NK cell BM homing. Given that untreated animals had about 20% AML cells in the BM when reaching pre-defined termination criteria, the role for myeloid leukemia-induced perturbations of the BM niche could simply not be studied in that model [28]. To better address the role of AML development in the present study, we here used the HL-60 cell line that similar to MOLM-14 establishes in the BM after i.v. inoculation before spreading to other organs but in contrast to MOLM-14 is less aggressive causing termination about 40-50 days from inoculation [17]. Importantly, in contrast to MOLM-14, HL-60 is relatively insensitive to NK cells, which is critical to our study as it avoids biased post-NK cell infusion analyses. However, this also implies that survival analyses comparing HL-60 engrafted mice treated with FUT7^+^CXCR4^R334X^ NK cells to those treated with control NK cells cannot be adequately done. While the present study establishes proof-of-concept with focus on the mechanisms for improved homing late in disease when AML-derived microenvironmental changes occur in the BM, future studies will have to address the therapeutic potential of this approach and how it for instance contributes to reduce tumor burden and improve survival when adoptive NK cell infusion is combined with for instance an AML-targeting BiKE or TriKE, chemotherapy prior cell therapy or when the NK cells are equipped with a CAR.

AML cells can create a self-reinforcing leukemic niche to support their survival per se but also chemoresistance [9–11, 24, 29–33]. While E-selectin is constitutively expressed on the endothelial cells of BM and skin [21, 22], stress conditions like cancer can induce E-selectin upregulation [9, 10]. Moreover, this is associated with angiogenesis and increased microvessel density [30, 34–36] that together with increased E-selectin expression is reported important for retention of leukemic stem cells in the BM [34]. In fact, the interaction between tumor cells and selectins has been proposed to positively correlate with disease development and is associated with metastasization [37]. Highly metastatic cancer cells often upregulate fucosyltransferases and present high levels of functional E-selectin ligands, leading to stronger interactions with E-selectin compared to the less metastatic subtypes. This has been reported for both solid tumors and hematological malignancies [38–41]. Further supporting the relevance of this axes for tumor development and progression is the fact that the percentage of E-selectin ligand expressing primary tumor cells in both MM and AML cells have been documented to be higher in relapsed patients compared to newly diagnosed patients [42–44]. Hence, these data highlight how central the E-selectin pathway is but at the same time suggest that reprogramming effector cells to harness this pathway for better tumor targeting may be a viable approach in certain settings for some cancer. Whether this strategy is equally good or better than disrupting the leukemic cell adhesion in the BM by inhibiting AML cell binding to E-selectin and by that mobilizing them from their protective niches remains to be addressed [11, 45].

Based on the reasoning above and our data showing that ex vivo expanded NK cells have low basal fucosylation levels resulting in poor adhesion to endothelial cells due to weak E-selectin binding, we in this study explored how to increase fucosylation levels with the goal of redirecting NK cells to the AML. Previous work has used ex vivo fucosylation of HSCs, CTLs and CAR-T cells to increase their BM homing capacity [23, 46, 47]. Here, we introduce the FUT7 enzyme by mRNA transfection, which has been reported superior [48], so that the NK cells can themself generate functional ligands able to better bind E-selectin on the vasculature in the AML niche. As presented in this paper, the introduction of FUT7 generated highly fucosylated NK cells for over a week with robust capacity to attach to activated endothelium under shear stress. Although they did not have significantly better BM homing potential when infused into healthy tumor-free mice with relatively few E-selectin^+^ BMECs, their ability to home to BM compartments of AML-bearing mice was more distinct. Since E-selectin binding contribute to the initial tethering/rolling phase, while chemokine receptor ligation is needed for the second phase to activate the cells and prepare for extravasation following firm adhesion [18], addition of CXCR4^R334X^ is important to add. Hence, although FUT7 introduction had a strong impact on NK cell BM homing in the context of AML, it was not enough to restore BM homing to the level observed for unmodified NK cells infused into tumor-free mice. Instead, the combination with CXCR4^R334X^ resulted in more prominent extravasation and increased infiltration of adoptively infused NK cells to the AML in the BM. This highlights the need for proper chemokine receptor signaling in addition to a proficient initial tethering/rolling phase. Although our data indicate the integrins needed for firm adhesion prior to extravasation are expressed by the NK cells explored in the current work, future studies may have to explore if these can be adjusted to ensure full extravasation potential towards the AML niche of the BM.

Another aspect that could be considered to further enhance NK cell homing to the AML BM is to combine adoptive transfer of FUT7^+^ CXCR4^R334X+^ NK cells with chemotherapy that, beyond cytoreduce, also help restore SDF-1α levels in the BM while further upregulate E-selectin [8, 22]. This could increase the potential of our approach further as overexpression of the CXCR4^R334X^ receptor has been documented to also work potently with normal SDF-1α levels [19]. Beyond this, one could also consider addressing the role of other chemokines and adhesion molecules such CCL3 and CXCL9/10 to promote infiltration into the proper anatomical location [29, 49, 50]. Indeed, the high upregulation of CXCR3 observed in our expanded NK cell product could be potentially relevant in this situation as well, since the levels of its ligands, CXCL9/10, have been shown to be elevated in the BM of AML patients [51]. Deciphering the AML-associated microenvironmental cues in more detail in relation to NK cell homing would allow to design NK cell products with a stronger capacity to infiltrate AML-rich compartments [29, 52]. This is not the least important for patients with chemorefractory disease but potentially also for patients with extramedullary disease that represents a unique challenge [53]. Depleting or blocking certain chemokine receptors could also be beneficial to avoid competition of NK cell infiltration into other non-AML engaged organs such as lungs, liver and spleen or their mobilization from the BM [20]. In relation to this, S1PR5 depletion could help in retaining NK cells within the BM, since the S1P concentration is elevated in the plasma of cancer patients [54] and is higher compared to the concentration in BM of AML patients [55].

That we cannot address tumor reduction and subsequent survival in the present model represents a potential limitation of our study. Moreover, mouse models per se comes with limitations. Despite relatively clear data showing the potential of capitalizing on CXCR4^R334X^ and introduction of FUT7, infusion of human cells into mice may inherently miss key data as the cross-reactivity of certain receptor ligand systems may not be complete. Nevertheless, the current model may serve as good grounds for assessing key insights. Future clinical exploration may better establish the therapeutic potential of the presented approach. Such studies may also shed light on data from individual patients with heterogenous disease rather than from genetically identical bred mice inoculated with an immortalized myeloid leukemia-derived tumor cell line.

In summary, our study presents the first evidence showing that AML development negatively impacts the ability of adoptively infused NK cell to infiltrate the BM. The data also reveal that genetic modification of NK cells to express functional E-selectin ligands and the GoF CXCR4^R334X^ can rewire their homing to the AML in vivo. Strategies to identify additional tumor-associated microenvironmental changes and how to capitalize on these to redirect adoptively infused cell products to the site of disease may be a promising approach to further improve NK cell-based immunotherapies of cancer. Future studies are warranted to address translational potential of this concept.

### Supplementary information

Supplemental Material

## Data Availability

For original data, please contact mattias.carlsten@ki.se.
