# Supplementary material for: Harnessing upregulated E-selectin while enhancing SDF-1α sensing redirects infused NK cells to the AML-perturbed bone marrow
Source: Leukemia. 2024 Jan 5;38(3):579–89. doi: 10.1038/s41375-023-02126-1 (PMC10912028; doi:10.1038/s41375-023-02126-1)
Supplement: Supplementary file 1 — Supplemental Material [file 41375_2023_2126_MOESM1_ESM.docx]

**Supplemental Material for**

**Harnessing upregulated E-selectin while enhancing SDF-1α sensing redirects infused NK cells to the AML-perturbed bone marrow**

Laura Sanz-Ortega^1^, Agneta Andersson^1^ and Mattias Carlsten^1, 2#^

^1^Center for Hematology and Regenerative Medicine, Department of Medicine, Huddinge, Karolinska Institutet, Stockholm, Sweden. ^2^Center for Cell Therapy and Allogeneic Stem Cell Transplantation, Karolinska Comprehensive Cancer Center, Karolinska University Hospital, Stockholm, Sweden.

^#^Corresponding Author: Mattias Carlsten, Karolinska Institutet, Medicinaren 25/Neo, HERM Floor 7, 141 83 Huddinge, Sweden. Phone: +46812380000. E-mail: mattias.carlsten@ki.se

**Supplemental methods**

*Cell lines and reagents*

The HL-60, K562 and MOLM-14 cell lines were purchased from ATCC (Manassas, VA, USA). The SMI-LCL cells were a kind gift from Dr. Childs, NIH, USA. The 721.221 WT cell line was a kind gift from Dr. Parham, Stanford, USA. The HUVEC cell line was purchased from ThermoFisher Scientific and cultured in Medium 200 supplemented with low serum growth supplement, as indicated by the manufacturer´s protocol. All the rest cell lines were cultured in complete media of RPMI 1640 (Gibco) supplemented with 10% FBS (Gibco).

*SDF-1α BM levels quantification*

BM supernatants from healthy (tumor-free), low and high tumor-bearing mice were collected and stored at -80°C until assayed. Mouse SDF-1α detection was assessed by the SDF-1α/CXCL12 Mouse ELISA Kit (Invitrogen). The BM supernatants were diluted to be in the linear range of the assay.

*Flow cytometry*

Cells were labeled with the following anti-human antibodies and reagents for flow cytometry. Anti-CD3 (UCHT1), anti-CD56 (NCAM16.2), anti-CD19 (SJ25C1), anti-CCR5 (2D7), anti-CCR7 (150503), anti-CD62L (DREG56), anti-CXCR1 (5A12), anti-CXCR4 (12G5), anti-CX_3_CR1 (2A9-1), anti-CD45 (HI30), anti-CLA (HECA-452), anti-CD11a (TS24), anti-PD-1 (MIH4), anti-NKp30 (P3015), anti-NKp46 (9-E2), anti-CD16 (3G8), anti-KIR2DL2/3D/S2 (DX27) and BD^TM^ CompBeads were purchased from BD Biosciences (San Jose, CA, USA). Anti-CXCR5 (FAB190A), anti-S1PR5 (282503), anti-KIR2DL/S1 (1127B) and anti-KIR2DL1 (143211) were purchased from R&D Systems (Minneapolis, MN, USA). Anti-CD44(IM7), anti-CD162/PSGL1 (KPL-1), anti-CCR1 (SF10B29), anti-CXCR3 (G025H7), anti-CD107a (H4A3), anti-LAG-3 (11C3C65), anti-TIM-3 (F38-2E2). Anti-CTLA-4 (L3D10), anti-CD96 (NK92.39), anti-NKG2D (1D11), anti-2B4 (C1.7), anti-KIR3DL1 (DX9) and the Zombie NIR™ Fixable Viability Kit and the TruStain FcX™ PLUS (anti-mouse CD16/32) antibody, were all purchased from Biolegend (San Diego, CA, USA). Anti-CD49d (HP2/1) was purchased from Novus Biologicals (Bio-Techne, Minneapolis, MN, USA). Anti-TIGIT (MBSA43) and the fixable dead cell marker LIVE/DEAD Aqua were purchased from Invitrogen eBiosciences. Anti-NKG2C (REA205) and anti-CD57 (TB03) were purchased from Miltenyi. Anti-NKG2A (Z199) and anti-KIR3DL1/S (Z27.3.7) were purchased from Beckman Coulter. Anti-LIR-1 (HPF1) was purchased from LS Bio (Seattle, WA, USA). The following anti-mouse antibodies were used: anti-CD45.1 (A39) and anti-CD62E (10E9.6) from BD Biosciences, and anti-CD31 (390) and anti-Lineage cocktail (anti-CD3ε(145-2C11), anti-Ly-6G/Ly-6C (RB6-8C5), anti-CD11b (M1/70), anti-CD45R/B220 (RA3-6B2), anti-TER-119/Erythroid cells (Ter-119)) from Biolegend. Flow cytometry stainings were performed at 4°C for 20-25 minutes, except for chemokine receptor stainings that were performed at 37°C for 25-30 minutes. The BD Symphony A5 Special Order Research Product (BD Biosciences) was used to acquire the samples, and data were analyzed using the FlowJo software (BD Biosciences).

*In vitro* *flow chamber assay*

*In vitro* adhesion assays under flow conditions were performed in a channel slide (µ-Slide I Luer, 0.4 mm height, ibidi), coated with HUVEC cells that were activated with TNF-α (20 ng/ml) overnight before the assay. 24-48 hours post mRNA transfection, non-modified and FUT7-modified NK cells were stained with Calcein-AM or CellTrace Calcein Red-Orange-AM (both from Sigma Aldrich) for 20 min at 37 °C in PBS. Non-modified and modified NK cells were alternatively stained in red or green in repeat experiments to exclude dye labelling effects. A 1:1 mixture of non-modified and FUT7-modified NK cells was prepared in PBS/0.5% FBS at a density of 2.5 × 10^5^ cells/ml. Flow chamber set-up was mounted on a Zeiss Axio Imager M1 Monitorized Fluorescence Microscope coupled with a HXP 120V lighting unit and PBS/0.5% FBS was infused at high flow rate to fill the system. Cells were infused at 0.5 dyne/cm^2^ (≈ 100 μl/min). The bright-field and corresponding 560 nm (green) and 645 nm (red) fluorescent images were recorded every 15 s for around 12 min. After that, the flow was stopped and cells were allowed to settle for 5 min, before restarting the flow and taking several images from different parts of the flow-chamber to assess final NK cell adhesion to the endothelium. Fiji Software (1) was used to generate the movies and for cell motility tracking.

*Degranulation assay*

mRNA or sham (no mRNA) electroporated NK cells and target cells were co-cultured at an effector-to-target (E:T) ratio of 1:1 for 1 hour at 37$^{\circ}$C. NK cells without target cells were used as negative control detecting spontaneous background activity. Following incubation, cells were stained with fluorescently conjugated antibodies for CD107a expression and markers to identify NK cells. ADCC was measured in co-cultures containing either 0.1, 1 or 10 μg/mL of rituximab (Roche, Basel, Switzerland), as indicated.

*Killing assays*

mRNA or sham (no mRNA) electroporated NK cells were co-cultured with target cells for 4 hours. Tumor killing was assessed using a Calcein-AM-based assay as previously described (2).

**References**

1. Schindelin J, Arganda-Carreras I, Frise E, Kaynig V, Longair M, Pietzsch T, et al. Fiji: An open-source platform for biological-image analysis. Nat Methods. 2012;9(7):676–82.

2. Segerberg F, Lundtoft C, Reid S, Hjorton K, Leonard D, Nordmark G, et al. Autoantibodies to Killer Cell Immunoglobulin-Like Receptors in Patients With Systemic Lupus Erythematosus Induce Natural Killer Cell Hyporesponsiveness. Front Immunol. 2019;10.

**Supplemental Figures and Figure legends**

**
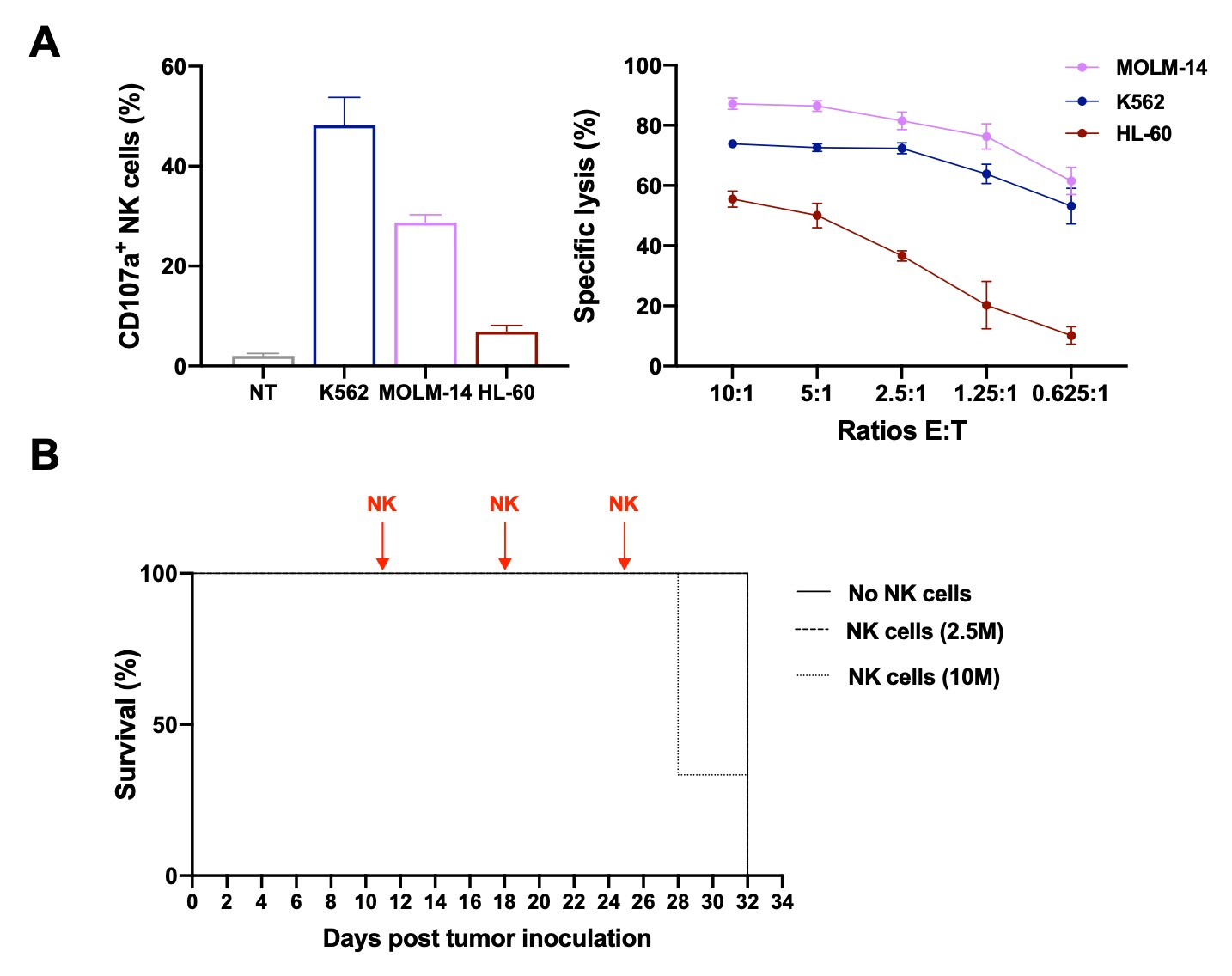
**

**Supplemental Figure 1. HL-60 has relatively poor *in vitro* and *in vivo* sensitivity to NK cells.** (A) NK cell degranulation and target cell killing following co-cultures with NK cells and the denoted target or without target (no target, NT) (n = 3). Bars, mean. Error bars, SEM. (B) Kaplan-Meier curve showing survival of mice after receiving intravenous injections of PBS (no NK cells), 2.5x10^6^ NK cells or 10x10^6^ NK cells once per week for 3 cycles starting 11 days post tumor inoculation (0.5x10^6^ HL-60 cells). Animals received 2x10^5^ IU of IL-2 intraperitoneally at the time of, and 24 respectively 96 hours after, each NK cell injection (n=3 mice/group).

**
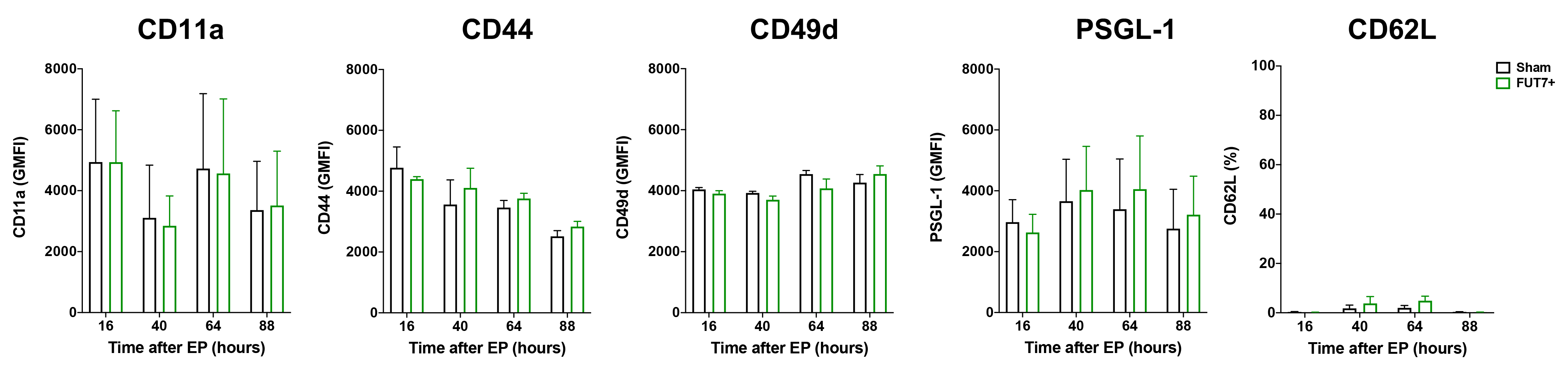
**

**Supplemental Figure 2. FUT7 mRNA electroporation do not alter the expression of other relevant adhesion molecules.** Expression of relevant adhesion molecules on ex vivo-expanded NK cells was assessed at different timepoints after electroporation with FUT7 mRNA. Sham (no mRNA) (n = 3). The Wilcoxon matched‐pairs signed‐rank test comparing mRNA electroporated vs sham electroporated NK cells was used for statistics.


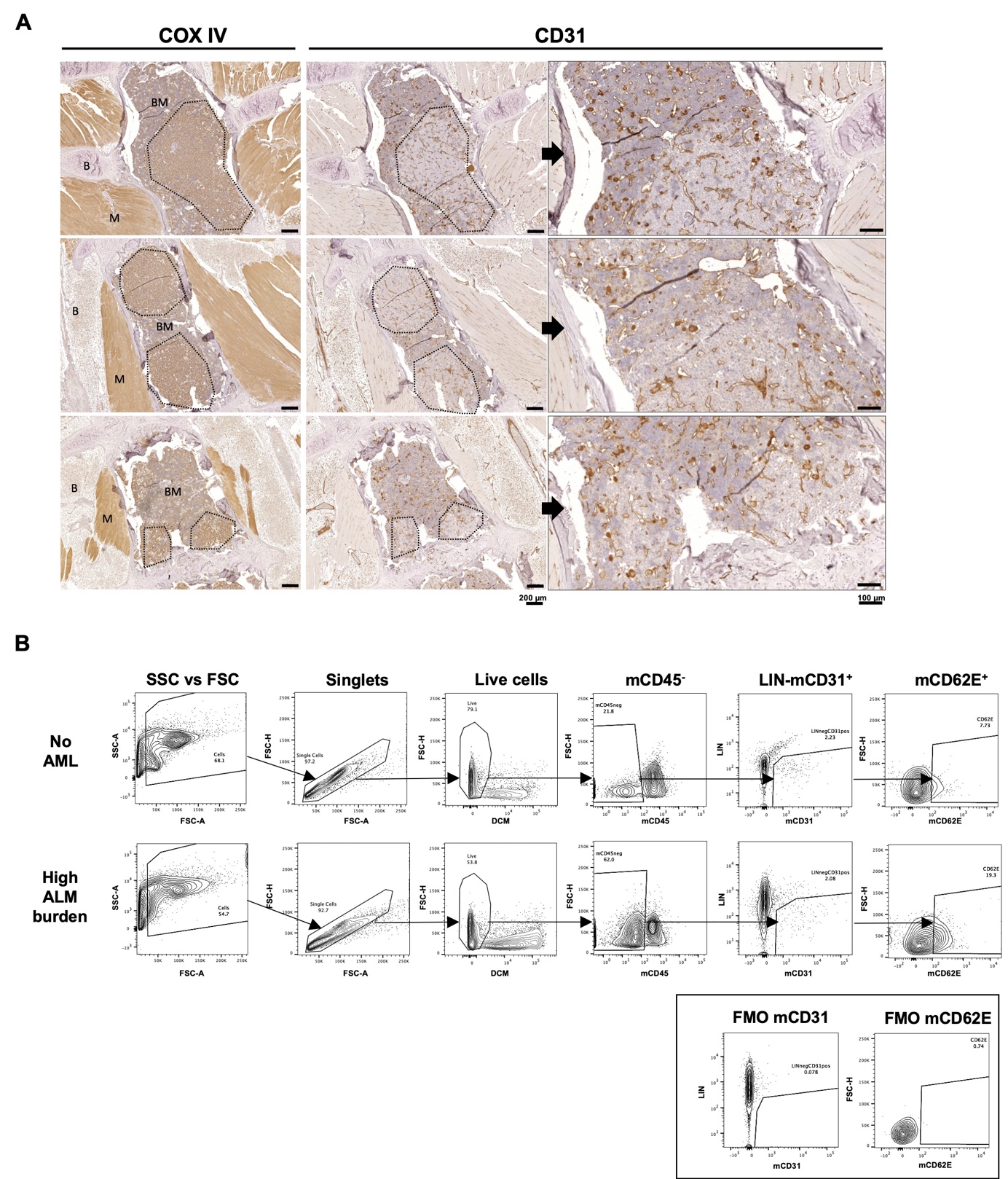


**Supplemental Figure 3. AML development induces morphological and phenotypic changes in the BM vasculature.** (A) Representative images showing AML BM niches in the AML (HL-60 cell)-inoculated NSG-SGM3 mice (identified by COX IV staining) colocalizing with changes in the pattern of vascularization (CD31 staining) within the BM. Dotted lines show AML regions (COX IV^+^). Scale bar: 200µm (left panels), 100µm (right panels). Legend: BM (bone marrow), B (bone) and M (muscle). (B) Gating strategy for BM endothelial cells (live^+^lineage^−^mCD45^−^mCD31^+^) and representative dot plots from one mouse in each group (healthy (no AML) vs high AML burden) showing E-selectin expression on BM endothelial cells. The corresponding controls (FMO) for CD31 and CD62E stainings are shown in the bottom panels.


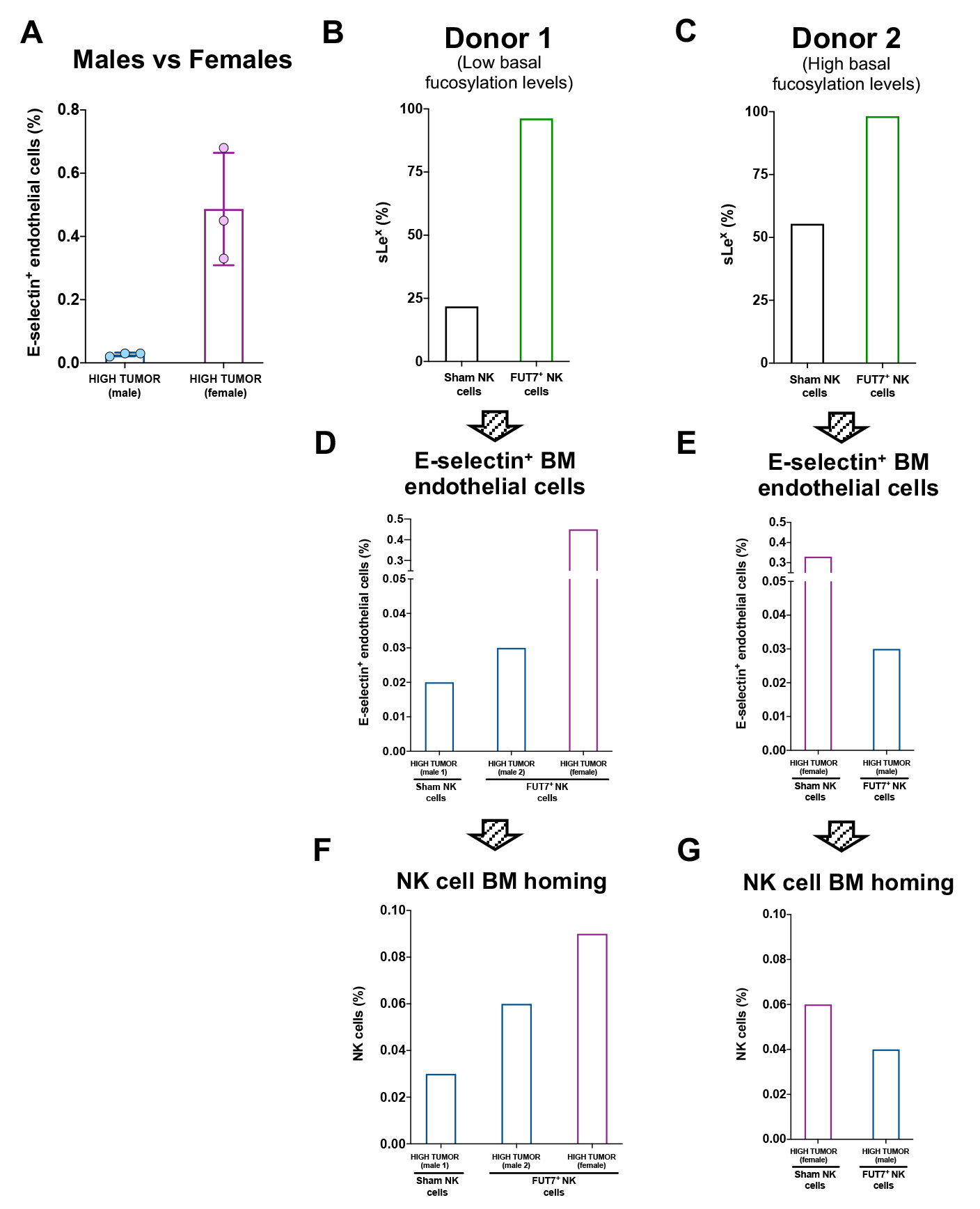


**Supplemental Figure 4. The need to introduce FUT7 to enhance BM homing seems to be determined by the basal fucosylation levels on expanded NK cells and the amount of E-selectin^+^ BM endothelial cells.** (A) Quantification of total E-selectin^+^ BM endothelial cells in (HL-60)-bearing mice with high tumor burden in the BM, comparing males vs females. Bars, mean. Error bars, SD. Fucosylation levels before and after electroporation with FUT7 mRNA of expanded NK cells from a donor with (B) low (donor 1) or (C) high (donor 2) basal fucosylation levels. Quantification of E-selectin^+^ BM endothelial cells in the recipient mice after transferring the non-modified (sham) or mRNA-modified (FUT7) expanded NK cells from (D) donor 1 or (E) donor 2 and (F), (G) the corresponding BM homing capacity of the transferred NK cells after 48 hours post injection.


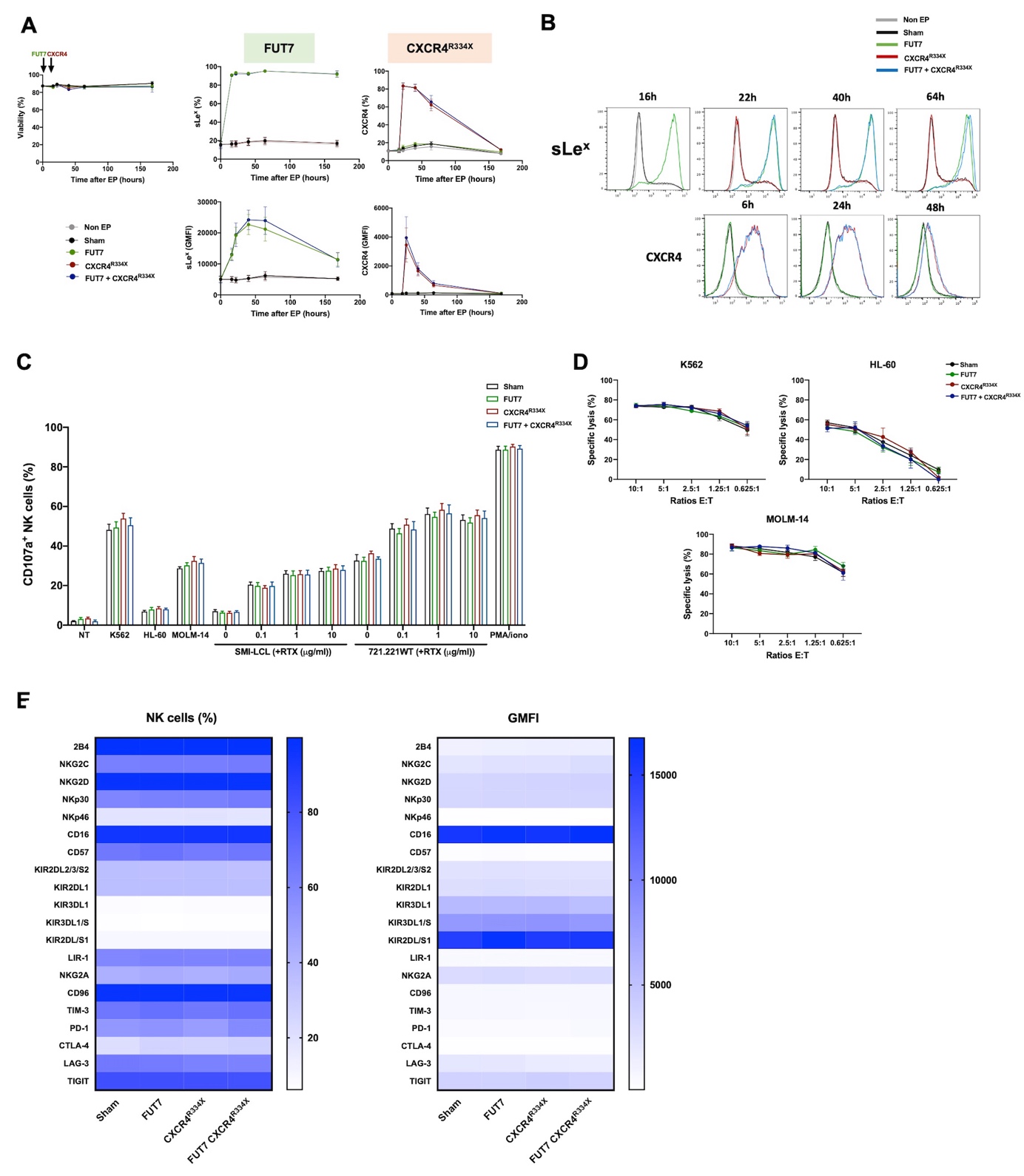


**Supplemental Figure 5. Human NK cells can be modified to co-express FUT7 and CXCR4^R334X^ while maintaining their viability, cytotoxic potential, and phenotype.** (A) Viability of ex vivo expanded NK cells from healthy donors following electroporation with 4 μg of FUT7 and/or CXCR4^334X^ mRNAs per million cells, and expression levels (% and GMFI) of the corresponding molecules. Sham (no mRNA) (n = 9-12). (B) Representative histograms of sLe^x^ and CXCR4 expression levels on expanded NK cells at different timepoints after mRNA electroporation with the corresponding mRNAs. Sham (no mRNA). (C) NK cell degranulation and (D) target cell killing following co-cultures with NK cells and the denoted target or without target (no target, NT) performed 20-24 hours after electroporation of the NK cells with FUT7 and/or CXCR4^334X^ mRNAs (n = 4-6). Bars, mean. Error bars, SEM. (E) Heat maps of protein expression (NK cells (%) and the corresponding GMFI) of relevant molecules (inhibitory and activating receptors, exhaustion markers) on ex vivo-expanded NK cells 20-24 hours after electroporation with FUT7 and/or CXCR4^334X^ mRNAs, assessed by FC. Sham (no mRNA) (n = 3). The Wilcoxon matched‐pairs signed‐rank test comparing mRNA electroporated vs sham electroporated NK cells was used for statistics.


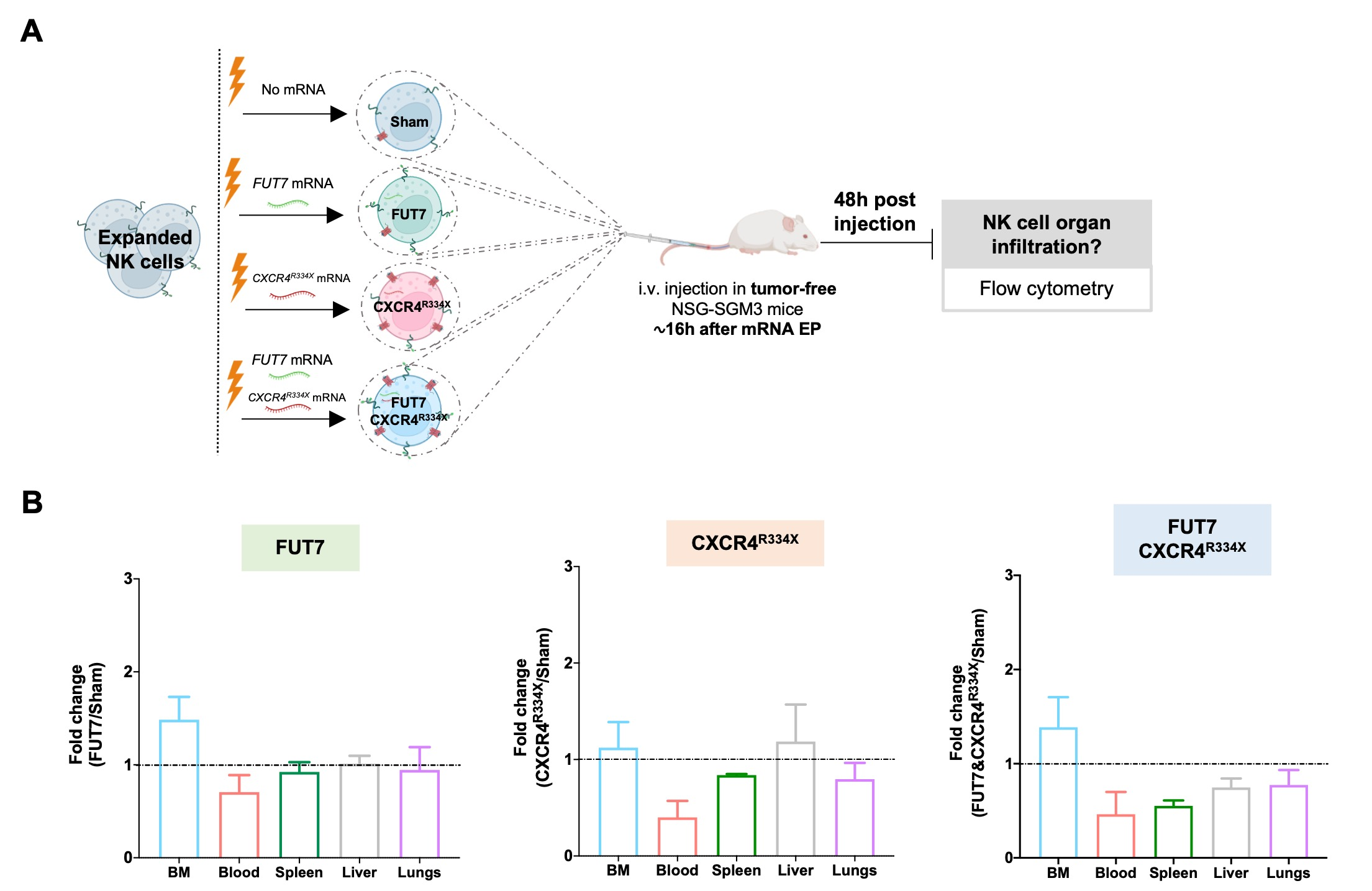


**Supplemental Figure 6. Introduction of FUT7 and CXCR4^R334X^ in expanded human NK cells does not lead to a significant BM homing in healthy mice.** (A) Experimental layout for in vivo homing of adoptively infused expanded human NK cells electroporated with mRNA coding for FUT7 and/or CXCR4^334X^ into healthy NSG-SGM3 mice. Created with BioRender.com. (B) NK cell infiltration in several organs 48h after cell transfer in terms of fold change relative to sham (no mRNA) condition, assessed by FC. Bars, mean. Error bars, SEM. The Mann-Whitney U test comparing mRNA electroporated vs sham electroporated NK cells was used for statistics.
